# Deciphering the RNA universe in sperm in its role as a vertical information carrier

**DOI:** 10.1093/eep/dvac011

**Published:** 2022-04-16

**Authors:** Miriam Kretschmer, Katharina Gapp

**Affiliations:** Department of Health Sciences and Technology, ETH Zurich, Laboratory of Molecular and Behavioral Neuroscience, Institute for Neuroscience, Winterthurerstrasse 190, Zurich 8057, Switzerland; Neuroscience Centre Zurich, ETH Zurich and University of Zurich, Winterthurerstrasse 190, Zurich 8057, Switzerland; Department of Health Sciences and Technology, ETH Zurich, Laboratory of Molecular and Behavioral Neuroscience, Institute for Neuroscience, Winterthurerstrasse 190, Zurich 8057, Switzerland; Neuroscience Centre Zurich, ETH Zurich and University of Zurich, Winterthurerstrasse 190, Zurich 8057, Switzerland

**Keywords:** sperm, epigenetic inheritance, RNA

## Abstract

The inheritance of neurophysiologic and neuropsychologic complex diseases can only partly be explained by the Mendelian concept of genetic inheritance. Previous research showed that both psychological disorders like post-traumatic stress disorder and metabolic diseases are more prevalent in the progeny of affected parents. This could suggest an epigenetic mode of transmission. Human studies give first insight into the scope of intergenerational influence of stressors but are limited in exploring the underlying mechanisms. Animal models have elucidated the mechanistic underpinnings of epigenetic transmission. In this review, we summarize progress on the mechanisms of paternal intergenerational transmission by means of sperm RNA in mouse models. We discuss relevant details for the modelling of RNA-mediated transmission, point towards currently unanswered questions and propose experimental considerations for tackling these questions.

## Intergenerational Effects at Odds with Classic Heredity

The Mendelian concept of genetic inheritance can only partly explain the inheritance of complex multifactorial neurophysiologic and neuropsychologic diseases. Pioneering research showed that post-traumatic stress disorder (PTSD) had a higher prevalence in offspring of parents who were Holocaust victims with PTSD independently of the known genetic predispositions [1, 2]. Similarly, the Dutch Famine study and its successors showed multiple metabolic diseases being prevalent in offspring and grand-offspring of mothers starving during the period of the Dutch Famine [3–5]. This suggests an impact of the environment psychologically and physiologically, not only limited on the generation directly exposed but also on their descendants, and thus a potential epigenetic constituent in the inheritance of complex diseases. The human studies mentioned above allude to an influence of environmental stressors across generations. Due to their descriptive nature and the complexity of the human environment that has to account for the socio-economic effects on the individual, they are limited in examining the mechanisms of epigenetic transmission. Animal studies allow a mechanistic interrogation since they can strictly control the environment throughout the entire life span of the individuals at stake. They can exclude genetic confounds by using inbred strains. The influence of psychological or metabolic stress has been previously studied extensively using rodent studies, building a foundation for research on the epigenetic inheritance of stress.

A variety of stressors can be effectively modelled by psychological or metabolic challenges in the environment. Exposures that have been used to model psychological and metabolic stress include acute social defeat, predators, immobilization, foot shocks or acute food restriction [6]. In addition, stress can be induced by pharmaceutical interventions like injections or inhalation of GABA antagonists, opioids, inflammation-promoting agents, components of the HPA axis and stress response like CRH or ACTH and their mimics, as nicely summarized in Patchevs’ review [7]. While corticosterone-mediated stress typically boosts physical performance [8], it can also have negative impacts [9–11], especially when chronic [12–14]. Not only does chronic stress disrupt specific psychological and metabolic functions in the subject experiencing it, but also in its offspring. Long-lasting alterations in gene expression and protein abundance caused by chronic stress have been found to be also transmitted to the progeny, resulting in impaired stress resilience as extensively described by Safi-Stibler and Gabory [15].

While effects of chronic stress on the offspring have been investigated extensively, researchers just recently started to look into the possible effects of acute stress not only on the individual but also on the offspring. In mice, males receiving a foot shock were mated to non-exposed females a few weeks later. The resulting offspring displayed altered body weight and glucose metabolism [16]. These results were confirmed to rely on germline-transmission by artificial insemination [17]. Acute paternal glucocorticoid receptor challenge influenced offspring molecular profile and altered glucose metabolism in mice [18]. In rats, males exposed to predator odours influenced not only maternal investment, affecting licking, grooming, retrieval and feeding, but also led to the development of anxiety-like behaviour in offspring [19]. In tree swallows (*Tachycineta bicolor*), brief corticosterone treatment simulating acute stress responses led to progeny being smaller [20].

Using both chronically and acutely stressed animal models, several mechanisms potentially underlying epigenetic transmission have been investigated. DNA methylation, histone modifications and RNA in the germline have primarily been explored as targets for parental signals as they have been proven malleable by the environment and experiences [21, 22]. The concept of epigenetic germline inheritance or meiotic epigenetic inheritance determines that the transmission of altered regulating epigenetic marks like DNA methylation, histone modifications and RNAs occurs through gametes, from one generation to their offspring [23]. Noteworthy, once induced, those altered regulators need to maintain their altered state in the gametes in order to deliver information about environment and experiences to the offspring. DNA methylation and histone modifications undergo epigenetic reprogramming during gametogenesis and in the pre-implantation embryo [24].

RNAs in the male germline are exempt from further reprogramming, making them an interesting target to investigate the mechanism of intergenerational epigenetic transmission from parental generation to their direct offspring. Obviously, intergenerational transmission is not limited to father-offspring effects but occurs conceivably even to a bigger extent between mother and progeny. This is because the oocyte contributes a larger amount of RNA to the embryo [25] and gestational signalling holds great potential to convey additional information to the growing embryo. Post-gestational maternal care, comprising not only nutrition but also licking and grooming, further represents a layer of potential influence on offspring health [26]. This richness of potential effectors of offspring health together with the very limited number of oocytes available per mouse complicates the study of female line effects. Regardless, while not the focus of our review, extensive research has also been conducted on epigenetic modifications and their function in epigenetic heredity in the oocyte, which is reviewed elsewhere [27, 28].

In cases where effects trespass not only the parent–offspring generation, but are further perpetuated to the offspring, they are often labelled as transgenerational, especially when the affected individuals are born from gametes that were not directly exposed to an environmental insult.

**Figure 1:**
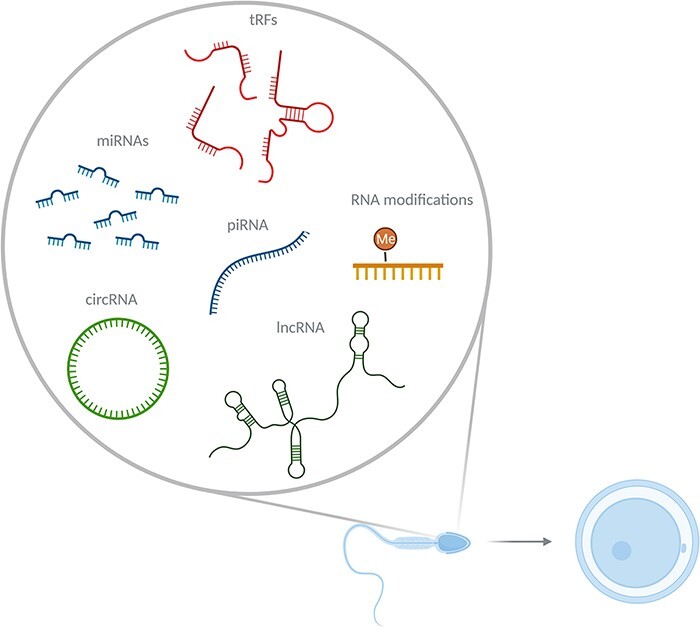
Sperm delivers a host of RNA to the oocyte during fertilization

Here, we will summarize findings on intergenerational transmission by means of sperm RNA, pointing out relevant subgroups of sperm RNA, (i) small non-coding RNAs, including (a) microRNAs (miRNAs), (b) transfer RNA (tRNA)-derived RNA fragments (tRFs) and (c) P-element Induced WImpy testis (PIWI)-interacting RNAs, and (ii) long linear RNAs and (iii) circular RNAs (circRNAs) (Fig. 1). We then explore key questions with regard to RNA-mediated inheritance. Finally, we suggest experimental considerations when testing these questions.

## Sperm RNA as a Vertical Information Vector

**Figure 2:**
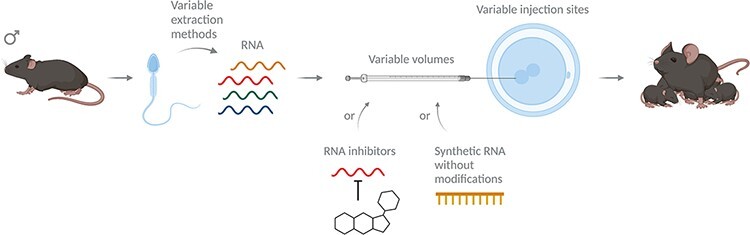
Methodological considerations of RNA injections into fertilized oocytes

Historically, the main experimental approach to causally test the effects of altered sperm RNAs on the offspring was sperm RNA injection into non-exposed fertilized oocytes [21] and examining offspring phenotypes in comparison to controls. In this crucial method, a superovulated female is mated with a wild-type male, and fertilized oocytes are extracted. At the 1-cell stage, when still both pronuclei are visible, RNA isolated from sperm of experimental or control males is microinjected into the male pronucleus. The embryos are then implanted into and fostered by pseudopregnant females to generate offspring. This offspring generated can then be investigated for its phenotype (Fig. 2). While it is a fundamental experiment in the field, the protocols used by different groups vary slightly, which can make direct comparisons between results difficult. Most groups inject RNA directly into the male pronucleus [29], but RNA can also be injected into the cytoplasm, and this is sometimes not specified [30, 31]. Furthermore, the amounts of RNA injected vary between labs, usually between 1 and 2 pl of a 0.5 [29, 32], 1 [31, 33], 2 [32, 34, 35] and 10 ${\mu}$g/ml [30] RNA solution. When injecting total RNA, RNA sizes might vary based on whether groups used TRIzol or column-based methods to purify RNA. Lastly, injections of synthesized RNA will by default lack potentially important RNA modifications [32, 34].

Interestingly, the first work exploring sperm RNA-mediated inheritance as an epigenetic mechanism using this method reaches back as far as 2006. In this study by the group of Minoo Rassoulzadegan, the offspring of animals heterozygous for a mutation in the *Kit* gene displayed white tail tips similar to their parents despite being of wild-type genotype, which could be traced back to altered Kit RNA levels and sizes in parental testis. When injecting total sperm or testis RNA of *Kit* mutant mice into non-mutant zygotes, the offspring displayed similar white tail tips as the mutant [30]. This provided the first evidence for a mechanistic involvement of sperm RNA in epigenetic germline inheritance. Since then, studies using that injection method and studies showing that specific sperm RNA species are delivered to the oocyte [36, 37], laid the foundation for a sperm to oocyte RNA signalling system to be postulated. Similar to the histone code, the makeup of the sperm RNA signal is suggested to determine the health of the offspring [38]. Sperm RNA differs substantially in its composition from RNA found in somatic cells and can be affected qualitatively or quantitatively by environmental influences [39–41]. How the sperm RNA signal is regulated by the environment in the first place and delivered to the offspring, and what challenges these questions are facing are discussed elsewhere in detail [22, 38, 42]. This review focuses on the findings on the specific subtypes of RNAs making up the sperm RNA signal in mice, small non-coding RNAs and their subtypes and long non-coding RNAs (lncRNAs), how these could influence the offspring and how to test their involvement in the transmission of phenotypes in a further refined way.

## Small Non-coding RNAs

### MicroRNAs

miRNAs are small non-coding RNAs, 21–25 nucleotides (nt) in size, which are involved in gene expression regulation in somatic cells of non-vertebrae and vertebrae. By engaging the RNA-induced silencing complex and the three prime UTR of mRNA, they lead to inhibition or degradation of mRNA [43]. miRNAs have been reported to direct DNA methylation in plants [44], yet there is no evidence for this in mammals yet. In sperm, it might seem surprising to find miRNAs given their transcriptionally rather silent state and low amounts of mRNAs.

Two controversial studies reported the necessity of sperm miRNAs for early embryonic development [45, 46]. In the context of environmental exposures, Rodgers *et al.* reported for the first time that males receiving 42 days of chronic variable stress throughout puberty or adulthood altered sperm small RNA composition measured with quantitative real-time polymerase chain reaction (qRT-PCR), where expression levels of nine miRNAs were altered [40].

Soon thereafter Gapp *et al.* [29] showed that total RNA purified from mouse sperm of fathers exposed to early life stress in form of unpredictable maternal separation and maternal stress (MSUS) in the first 2 weeks of life persistently affected behaviour and metabolism in offspring generated from RNA injections into fertilized non-exposed oocytes. Using RNA-seq, said RNA showed alterations in a payload of 43 miRNAs in sperm, 5 of which were confirmed by quantitative PCR (qPCR) in sperm, brain and serum [29]. One of the confirmed miRNAs, miR 375, was also found to be altered in a study using a chronic stress model by Rodgers *et al.* [40]. Gapp and colleagues concluded that the altered sperm miRNA effectively transmitted the phenotype to the offspring [29]. Around the same time, injection of nine miRNAs previously identified to be elevated in sperm of mice exposed to chronic variable stress [40] into the zygote led to dysregulated stress responses in the resulting offspring, with reduced corticosterone response and reduced gene expression levels of a set of genes important for the response HPA axis [47].

A relation between sperm miRNA and transmission of phenotype in the offspring could not only be made for traumatic stress-induced behavioural changes, but also for dietary stress–induced metabolic changes. High-fat diet is commonly used to introduce metabolic stress. In comparison to standard chow, animals are fed a diet with significantly higher digestible energy by providing butterfat in the diet. Grandjean *et al.* used such a high-fat diet, feeding it to males 3 weeks of age for 4 months. At an age of 16 weeks, 13 miRNAs, as of RNA-seq, were elevated in sperm and testis in animals displaying the metabolic phenotype, with higher body weight and blood glucose levels. They could confirm five of those miRNAs by qRT-PCR, none of which overlapped with the miRNAs altered in the studies of Rodgers *et al.* [40] and Gapp *et al.* [29]. Microinjection of those miRNAs into a wild-type zygote led to similar metabolic alterations in the resulting offspring [31]. It had previously been shown that a high-fat diet, using same amounts of fat as Grandjean and colleagues, caused alterations in testes and sperm miRNA payload in microarray, confirming with qPCR 11 miRNAs of those to be altered in testes, 4 of which in sperm. Progeny sperm RNA content was not examined, although F1 and F2 progeny showed the paternal obese phenotype and insulin resistance while being fed a standard diet [39]. One of the paternal miRNAs demonstrated to be altered, miRNA 340-5p, has been reported to be altered in the high-fat study by Grandjean and colleagues [31].

Further confirming a causal relation between sperm miRNA and epigenetic transmission to the offspring, a study showed that the effects of transmitted miRNA could be reversed with a specific miRNA inhibitor. Adult male mice were exposed to environmental enrichment, enhancing their synaptic plasticity and cognition tested for contextual fear-conditioning and in the Morris water maze paradigm. Their sperm displayed altered levels of miR212 and miR132 as of qPCR, none of which overlapped with the studies by Rodgers *et al*. [40], Fullston *et al.* [39], Gapp *et al.* [29] or Grandjean *et al.* [31]. The phenotype was also apparent in their offspring generated with natural breeding and could be counteracted by co-injection of an inhibitor to miR212/132 [48]. While the transmission of beneficial effects of enriched environment to the offspring had already been suggested before [49, 50], this was the first time this could be conclusively related to alterations in miRNAs in sperm.

Finally, the contribution of miRNAs in transmitting effects of chronic stress to the offspring has been corroborated by Wang *et al.* [51] in a study that blocked the transmission of the stress-induced phenotype by injecting a pool of miRNA inhibitors. Males aged 8 weeks were exposed to unpredictable mild stresses daily for 5 weeks, resulting in a depression-like model as tested with a forced swim test and a sugar preference test. RNA-seq and qPCR revealed altered levels for 17 miRNAs in their sperm. Directly after the stress paradigm, sperm small RNA was isolated and injected into non-exposed zygotes to generate offspring. At 2 months of age, the offspring was susceptible to depression-like symptoms but did not display altered small RNA levels in sperm. Injecting zygotes that had been generated with sperm of males that underwent the unpredictable mild stress with antisense strands to neutralize miRNA rescued the phenotype [51]. Thereby, the role of miRNAs in transmitting the effects of chronic stress can now be judged as fairly established.

While the above-mentioned studies all point towards a classical miRNA-induced regulation of mRNA targets in the early embryo, it cannot be excluded that miRNA changes in sperm primarily affect mRNAs in sperm cells, a class that will be discussed further down.

### tRNA-Derived RNA Fragments

Importantly, the analysis of total sperm RNA showed that miRNAs do not constitute the largest portion of small non-coding RNAs, but instead tRFs [52]. TRFs in sperm are derived from the 5ʹ and 3ʹ ends of tRNAs and are distinctly bigger in size than miRNAs with 29–34 nt. In somatic mammalian cells, tRFs are involved with Argonaute proteins to promote cleavage of sequence matched targets [53]. Sperm tRFs are proposed to regulate genes linked to retroelements in the early embryo [32]. Sharma *et al.* [32] measured the abundance of tRFs in epididymal sperm with small RNA-seq, five of which were upregulated in adult males that were fed a low protein diet consisting of 10% instead of 19% protein. Their *in vitro* fertilization-derived offspring displayed upregulated expression for biosynthesis-related genes. They found tRFs in paternal testes not to be affected, yet epididymal tissue also displayed the same changes in tRF population, from which they concluded that altered tRFs in sperm were not derived during spermatogenesis. Based on coincubation experiments of sperm cells and epidydimal epithelial exosomes followed by comparative small RNA sequencing, they reasoned that at least a large proportion of them are provided to spermatozoa by epididymal epithelial cells via extracellular vesicles during epididymal transit from caput to cauda. They further provided evidence for tRFs functioning in sperm as regulators for genes linked to the endogenous retroelement MERVL, a murine endogenous retrovirus [32].

Corroborating tRF uptake during epididymal transit, Gapp *et al.* [18] demonstrated that one tRF was unaltered in the caput epididymis of adult males 3 h after injection of dexamethasone, a glucocorticoid receptor agonist, yet altered in cauda epididymis using a combination of small RNA-seq and qRT-PCR. They further proposed that upregulation of tRF Arg-CCT-2 in cauda epididymal sperm was potentially derived from serum-circulating exosomes, which also showed tRF Arg-CCT-2 upregulation [18].

Also using a high-fat diet mouse model like Grandjean *et al.* [31] for their study on miRNAs, Chen *et al.* showed even earlier that such a diet in male mice altered tRFs abundance and RNA methylation levels (see in the section on RNA modifications). When injecting total sperm RNA or only sperm tRFs from males fed this diet into a wild-type zygote, the resulting offspring developed metabolic disorders similar to their fathers while injection of miRNA or long RNA fractions resulted in no effect [34]. This study by Chen and colleagues together with the one by Sharma *et al.* [32] provided the first causal evidence of sperm tRFs transmitting acquired information to the offspring.

Furthermore, a study by Cropley *et al.* described altered sperm RNA—especially tRFs—in F1 offspring of prediabetic males. When mated with wild-type females, the resulting F2 offspring showed similar changes in glucose metabolism as their F1 fathers with the phenotype only wearing off in the F3 generation [54]. Another study confirmed the role of tRFs in epigenetic transmission, by injecting total RNA or tRFs, but not larger RNAs (sized 40–90 nt) isolated from sperm of males with a high-fat diet-induced hedonic and metabolic phenotype into fertilized egg cells with the resulting progeny displaying the same phenotype. Based on their results together with the findings of Sharma *et al.* [32], they speculated that tRFs transmit acquired information via the regulation of MERVL elements in the early embryo [32, 35].

Overall, the above studies clearly demonstrate the responsiveness of tRFs to environmental, in their majority dietary, challenges and their involvement in the transmission of non-genetically transmitted phenotypes.

### PIWI-Interacting RNAs

Besides alterations in miRNA, Gapp *et al.* [29] also described changes in one cluster of PIWI-interacting RNAs (piRNA) in sperm of males exposed to early life stress in form of MSUS in the first 2 weeks of life. piRNAs are small non-coding RNA (26–31 nt) involved in the regulation of gene expression by interacting with the PIWI subfamily of Argonaute proteins [55]. In the brain, they facilitate the methylation of promoter regions to enhance synaptic plasticity [56]. In the germline, prepachytene piRNAs are similarly involved in the methylation of genomic sequences harbouring transposable elements to stabilize the germline genome [55], whereas pachytene piRNAs and post-meiotic spermatid piRNAs contribute to post-translational mRNA cleavage [57, 58].

A concomitant change in piRNA and tRFs requires consideration with interpreting RNA injection results, since sperm RNA injections using size selected fractions of total sperm RNA cannot distinguish piRNAs from tRFs. Hence, Gapp *et al.* [29] could not exclude a contribution of piRNA alterations to the transmission of effects of MSUS to the resulting offspring [29]. While Sharma *et al.* and Chen *et al.* did not comment on piRNA changes [32, 34], Grandjean *et al.* also noted changes in piRNA expression in their study [31]. Males that were fed a high-fat diet had elevated piRNA levels in their spermatozoa for 63 different piRNA clusters [31]. While environmental influences seem to regulate piRNA payload, the role of piRNAs in epigenetic germline inheritance remains to be elucidated. piRNAs are crucial to chromatin remodelling [59] and retrotransposon silencing [60]. A recent study demonstrated that fertilization of wild-type oocytes with sperm harbouring a homozygous deletion of a specific piRNA cluster on chromosome 6 resulted in abnormal heterozygous embryos with reduced embryonic survival [61]. This was attributed to regulatory functions of piRNAs on mRNAs prior fertilization but could well have also a component relevant post-fertilization. On the other hand, a study by Yuan *et al.* found that paternal pachytene piRNAs are not required for persistent fertility in mice lacking MIWI in round spermatids [62]. In non-vertebrae, piRNAs have been clearly involved in epigenetic inheritance as is discussed elsewhere [22].

Convincing proof for the involvement of piRNAs in epigenetic inheritance needs yet to be established in mammalian systems.

## Long Linear RNAs

In contrast to the small RNA species discussed so far, we define long linear RNAs as RNAs >200 nt. It comprises both coding mRNAs as well as lncRNA. The latter can be subclassified as sense, antisense, bidirectional, intronic, and intergenic lncRNAs. They are involved in a host of functions of gene regulation concerning chromatin structure and factor recruitment in somatic cells, as summarized elsewhere [63]. However, they are equally involved in regulating gene expression in sperm where they modulate spermatogenesis [64]. As described above, studies on the effect of diet-induced transmission, using a high-fat diet, were unable to phenocopy effects in the offspring by sperm lncRNA injections [34, 35]. Whether the long RNA fraction was affected in those models had not been assessed. Different so for a model of early trauma, consisting of exposing males to maternal separation and unpredictable maternal stress in the first two weeks of life: these males displayed considerable changes in their sperm long RNA payload, assessed by next-generation sequencing, including alterations in transposable element abundance [65]. Although not specifically investigated, this could be related to changes in sperm piRNAs observed in the same model. Typical behavioural changes in increased risk-taking and behavioural despair and metabolic changes in glucose response were partially mimicked by injection of sperm long RNA fraction. Interestingly, injection of small RNA fraction was sufficient to copy increased behavioural despair. Gapp and colleagues interpreted that both lncRNAs and small RNAs were necessary to transmit all behavioural and metabolic alterations to the offspring yet concluded that sperm long RNAs are functional [65].

A recent study addressed a long-standing controversy around sperm long RNA, that is whether cloned mRNA fragments might represent remnants and would not reflect full-length functional RNAs. Previous analysis used bioinformatic tools to establish whether the read fragments at least generally cover the full-length RNAs in question. A new study uses single-molecule long-read sequencing to unambiguously determine the presence of intact RNA molecules in mature sperm [66]. Such sequencing will in the future certainly prove useful also in the context of the assessment of changes following an exposure.

In conclusion, surprisingly little research has been conducted on environmental effects on long linear RNAs in sperm despite their potential of transposable elements to translate into genomic changes and thereby inducing long-term more stable consequences. It will be interesting to see whether this will change given the new technical possibilities to determine intact long RNA and the growing certainty that those are indeed present in mature sperm.

## Circular RNAs

To add yet another interesting class of sperm RNAs to the long list of mobile molecules, Gapp and colleagues recently reported for the first time changes in circRNA in response to a strong stress mimic in the form of a dexamethasone injection [18]. circRNAs are circular long RNAs, 500–4000 nt in size, that are produced by a form of alternative splicing called back splicing. During this event, the 5ʹ and 3ʹ end of a pre-mRNA are covalently linked. They are particularly interesting since they are highly stable in comparison to linear counterparts [67, 68] and could thus be protected during sperm to oocyte delivery and affect offspring embryonic phenotype. Many regulatory functions have been described for circRNAs in somatic cells, ranging from regulation of pluripotency and differentiation to control of proliferation [69]. CircRNAs have been demonstrated to be very highly expressed in testis [70]. In the context of epigenetic inheritance, three specific mechanistic properties stand out. First, circRNAs have been shown to be stored in the maturing germline when transcription ceases as templates for the translation of peptides during later stages of spermatogenesis [71]. Upon delivery to the oocyte, they could also potentially engage in peptide translation. Second, one very hotly debated role concerns the sponging of miRNAs. Such was shown conclusively for Circ *SRY* that has 16 mir138 binding sites [72]. Hence, if transmitted to the oocyte, circRNAs could act as miRNA sponges, thereby regulating targets of miRNAs in the offspring embryo and amplifying effects on gene expression. In the study by Gapp *et al.* [18] data from single 2-cell embryo expression indeed point towards such regulation [18]. Sat1, a mRNA target of the miRNA that has predicted binding sites on both circRNAs, that are upregulated in sperm, was shown to be upregulated in the 2-cell embryos, suggesting a sponging of the regulatory miRNA. Lastly, circRNAs were suggested to function as protein decoys, which prevent or promote the interaction of proteins with binding partners [73] and may support or repress their mobility between cytoplasm and nucleus [74]. This last mechanism could influence epigenetically transmitted proteins in their function or target proteins translated in the early embryo.

Overall, circRNAs show great potential to amplify intergenerational signals in the early embryo and are therefore a target worthwhile considering.

## RNA Modifications

The transmission of diet-induced effects was suggested to be reliant on the changes in RNA modifications [75], building on earlier results from the group of Minoo Rassoulzadegan. Her results had shown that a knockout of the tRNA methyltransferase Dnmt2, which engages tRFs as its substrate, prevented the *Kit* mutant phenotype from being transmitted to the offspring. Microinjection of sperm RNA from *Dnmt2^−/−^* males into non-mutant zygotes did not result in the *Kit* mutant phenotype in the resulting progeny [33]. Chen’s findings corroborated this, in that a deletion in *Dnmt2* prevented the transmission of a high-fat diet-induced metabolic phenotype by sperm RNA and was associated with altered sperm RNA expression, especially tRFs, and prohibited the rise in RNA modifications they observed in high-fat diet mice. They concluded that those modifications and altered levels were necessary to compose the sperm signal for the transmission of paternal acquired information [75]. This stands in contrast to the results obtained by Sharma *et al.* [32] when injecting synthesized tRFs mimicking those tRFs that showed changes upon altered diet. They were able to copy the paternal low protein diet-induced gene expression changes in the early embryo [32]. This could indicate that each study was capturing distinct aspects of a complex phenotype and demonstrates the potential of plural interpretations.

A new preprint by Jung *et al.* [76] on the effects of bisphenol A (BPA) exposure on intergenerational obesity also emphasizes the involvement of chromatin changes at the *Fto* gene, encoding a N6-methyladenosine (m6A) methyltransferase, leading to decreased m6A levels. Genetic deletion of a transcription factor binding site at the *Fto* gene abolishes the transmission of obesity [76]. This again points towards a critical involvement of m6A. RNA hypomethylation has been implicated in increased recruitment of enhancer RNAs to DNA thereby altering chromatin accessibility and transcription [77], but simultaneously the binding might confer a stability advantage during fertilization as to influence early embryonic transcription. Furthermore, m6A levels have been shown crucial for circRNA biogenesis during sperm maturation [71], potentially indicating a complex interplay of transcription factor-mediated chromatin looping, non-coding RNAs and RNA modifications in the transmission of regulatory signals of gene expression that perpetuate the effects of BPA across generations.

Altered RNA modifications have also been reported for other types of exposures apart from diet. Depressive patients challenged with glucocorticoids and mice exposed to stress show a tight regulation of m6A and N6,2ʹ-O-dimethyladenosine in the brain [78]. That m6A levels of mRNAs might be also affected in the germline of stress-exposed males is to be expected but remains to be determined.

RNA modifications are also relevant for technical reasons. Certain modified bases can lead to lower cloning efficiencies and bias the assessment in genome-wide sequencing approaches. A recent preprint by tRF experts from the same group that first reported the importance of tRFs in transmission benchmarked different sequencing methods and concluded conventional small RNA sequencing library preparation methods to be inaccurate for tRF profiling due to their high degree of modifications [79]. This prompts reconsideration of prior studies on the contribution of tRFs. Luckily, novel technologies embrace the potential discrimination of certain modified RNAs [80] and will hopefully accelerate the elucidation of the effects of environmental exposures on RNA modifications and their contribution to intergenerational phenotypes.

Taken together, it is clear that several RNA classes and their modifications are contributing to epigenetic germline inheritance. The complexity of the sperm RNA payload calls for investigation on the origin of sperm RNA. Furthermore, it will be crucial to determine which of the presented RNA classes are indeed delivered to the oocyte, and whether this changes in response to environmental exposures. The mechanisms underlying such transmission might bear some clues on how a given RNA excerpts its function.

## Where Does Sperm RNA Come from?

The transcriptionally silent state of mature sperm poses an obvious question. Where or when is mature sperm RNA transcribed? Early studies explored the possibility of mobile RNA. Sperm RNAs might originate from somatic cells elsewhere in the body and get taken up by sperm cells. This was tested by xenografting tumour cells to germline distal sites. Exogenous tumour RNA, being technically more distinguishable from endogenous sperm RNA, could be detected in sperm and thereby provided the first proof of principle [81]. Later studies by Sharma *et al.* [32] revisited the idea of exosomal delivery of tRNA fragments in a more natural setting focusing on epididymal exosomes, then termed epididymosomes [32]. With the advent of novel RNA-labelling techniques such as SlamITseq [82], Sharma provided data on the presence of metabolically labelled epididymal RNA in sperm [83]. This RNA was presumably taken up during the transit from caput to cauda epididymis and focused on miRNAs as opposed to tRNA fragments. A disputed follow-up study claimed that these acquired miRNAs were required upon delivery to the oocyte for embryonic development [84]. The importance of epididymal miRNA contributions for the transmission of the effects of chronic stress has been proposed by a study from the Bale lab [85]. They mimicked the transmission of effects to the offspring by injections of sperm incubated with epididymosomes from stressed males, suggesting their importance in a natural mating setting of chronically stressed fathers. The sperm RNA payload however likely also contains remnants of prior transcription as has been shown for circRNAs [71] and tRNA fragments [18] and suggested for long RNAs [86].

## How and Where Are RNA Molecules Functional?

As we explore which of the sperm RNA subgroups is relevant for epigenetic germline inheritance, it is also important to investigate which of these are effectively transmitted to the oocyte. The standard method for exploring the effects of a certain sperm RNA type has been to isolate total sperm RNA and to inject it or RNA fractions into non-exposed fertilized oocytes. This provided information about the functionality of sperm RNA, but not the transfer from sperm to oocyte itself. So far, sperm RNA delivery has been inferred from comparisons of microarray or sequencing data from unfertilized and fertilized oocytes [37, 87, 88]. The lack of statistical comparisons makes them prone to artefacts introduced by for instance divergent sequencing depth or sample quality. Metabolic labelling methods such as mentioned above in the context of exosomal RNA delivery [82, 83] could instead be employed to unambiguously establish which RNA classes are transmitted.

The prime curiosity remains RNA fragility. How can RNA retain sufficient stability? As alluded to in the prior section, circRNAs would have a clear advantage over free linear RNAs and therefore make them a likely relevant candidate. To experimentally approach their potential ability to sponge miRNAs in the embryo, such miRNAs could be provided in excess to counterbalance the circRNA-induced reduction. This reversal would expectedly lead to a downregulation of mRNA targets of said miRNAs and thereby normalize the circRNA-induced upregulation of mRNAs.

## DNA-Bound RNA

In opposition to inherently stable circRNAs, linear sperm RNA subgroups most likely rely on certain stabilization to transfer information to the offspring. It is well recognized that some RNA is bound to DNA [77]; however, experimental procedures discussed here so far with the exception of the study from Jung *et al.* [91] ignored this fraction.

Chromatin-associated RNAs have been described as highly stable structures [89], revealing that there are *cis*- and *trans*-interacting RNAs. *Cis*-interacting DNA-bound RNAs stay on site of their transcription, whereas *trans*-interacting DNA-bound RNAs leave their site of synthesis to interact with another genomic locus [90]. Western blot showed the presence of RNA polymerase II in mature sperm [91], a remainder of active transcription during spermatogenesis. As evidenced by findings of the group of Victor Corces, a small fraction of stalled nascent RNA appears bound to chromatin in spermatozoa. This RNA could possibly remain on site and thus form a *cis*-interaction with its DNA template. Given the evidence that spermatozoa receive a majority of their RNA content from the epididymis via epididymosomes [42], a *trans*-interacting mode is expected as to how RNAs can associate with sperm DNA. *Trans*-interactions can occur directly via DNA:RNA hybrids or indirectly via a mediating RNA binding protein where they are described to regulate transcription [92]. *Trans*-interacting RNA binds directly to DNA by forming triplex structures. By Hoogsteen base-pairing, RNA binds via hydrogen bonds to the major groove of the double-stranded DNA, winding around the double helix [93, 94]. This leads to stabilization of the RNA [95] and allows it to guide transcription regulators to distinct sites, best experimentally proven so far with lncRNAs [93].

A multitude of chromatin enriched lncRNAs have been found in adjacency to active genes [96] and have later been confirmed as transcriptional co-activators [97]. The insights on specific lncRNAs creating triplex formations and their implications for genomic regulation are reviewed elsewhere [93]. Interestingly, there has been recent evidence that precursor mRNA might also function as regulatory lncRNA. Skalska and colleagues proposed in 2017 that precursor mRNA functions similarly to non-coding RNAs by forming transcription hubs around their site of transcription to regulate gene expression [98]. Regarding the high abundance of protein-coding RNAs in the DNA-bound RNA fraction of the presented data, it might be possible that some of these are functioning as expression regulators, as suggested by Skalska *et al.* and Wei *et al.* [98, 99]. This possibility needs to be investigated further, as the understanding of DNA-bound RNAs in sperm is very rudimental. lncRNA content in sperm could be analysed by nanopore sequencing to account for the transcript lengths and compared with previous findings [96, 97]. Furthermore, to confirm these lncRNAs are bound to sperm DNA directly, they should be compared after mapping with results from a genome-wide characterization of DNA:RNA triplex structures [100]. Alternatively, chromatin isolation by RNA purification [101] or mapping RNA–genome interactions [102] might be employed to discover and examine DNA-bound RNA. In a follow-up, the question of whether this specific fraction bears any advantage during sperm to oocyte transmission could be explored. The characterization of lncRNA content and associated structures in the zygote after mating or *in vitro* fertilization, in comparison to the non-exposed oocyte, might prove useful to test such hypotheses.

Similar to lncRNAs, several miRNAs have been shown to bind double-stranded DNA by forming triplex structures. Bioinformatic analysis investigated triplex-forming binding sites for miRNA. The sites were enriched in genes that positively correlated their expression with miRNA expression of the miRNAs binding those sites [103]. Similarly, a recent *in silico* study compared genomic sites where miRNAs bind with transcription factor binding sites in the genome. It revealed conserved motifs in miRNA transcripts and predicted them to bind specific DNA sequences [104]. Other research so far covered the indirect binding of miRNAs with DNA via Ago2 protein [105] or triplex formation miRNA-mediated detection [106, 107]. Experimental evidence for miRNAs binding directly to genomic DNA by the formation of triplex structures is still lacking to confirm the *in silico* predictions. If indeed specific triplex-forming miRNAs can be proven experimentally, this adds to the idea that sperm RNAs—in this case miRNAs—might benefit from stabilization by the formation of triplex structures.

Two studies suggest tRF binding to DNA via Ago, with an impact on gene expression [108, 109]. While these studies indicate an indirect binding to the DNA, it is currently unclear whether tRFs also engage in direct DNA binding.

Similarly, piRNAs most likely bind directly or indirectly to DNA as to induce DNA methylation and gene silencing [110, 111].

The most widely studied DNA-binding RNA is telomeric repeat–containing RNA (TERRA). Interestingly, TERRAs bind to DNA by forming R-loops, opposing to the triplex formation of the RNAs described above. R-loops are formed by the DNA double helix opening up and the RNA attaching to one of the DNA strands. TERRAs are involved in telomere maintenance at chromosome ends [112, 113]. They form G-quadruplex structures, and *in vitro* studies indicate that co-binding of the human protein FUS to this structure and DNA quadruplexes at telomeres could recruit epigenetic modifiers and thereby regulate heterochromatin formation [114]. Furthermore, DNA G-quadruplexes in gene bodies have also been observed to help resume transcription [115], or opposingly to stall transcription by facilitating the stabilization of nascent transcripts into R-loops [116]. Alternatively, the formation of DNA–RNA quadruplexes might terminate transcription of that locus altogether [117, 118]. This rather resembles the *cis*-interacting mode of RNA binding DNA [90].

Not only the stabilization of RNA by the formation of triplex structures with the DNA double helix and its delivery have to be proven relevant for epigenetic inheritance. The mechanism of action post-fertilization also requires further investigation. Detailed intersection of sperm RNA data, DNA Pol II data and genome-wide characterization of DNA:RNA triplex structures [100] and DNA quadruplex structures will likely reveal interesting further indications on the role of DNA–RNA interactions in sperm.

## Experimental Considerations

Assuming that paternal sperm RNA bound to DNA is favoured in the transmission, two mechanistic scenarios are to be expected post-fertilization. (i) The RNA is released to find complementary specific sites on the maternal chromosomes and/or to regulate RNA transcripts post-transcriptionally. (ii) The RNA stays bound to the paternal allele and continues to regulate gene expression monoallelically in an imprinting-like fashion [119, 120].

It is important to mention that sperm RNA injections of exposed males into fertilized non-exposed oocytes are not suitable to assess the potential contributions of DNA-bound RNA. When RNA is harvested for such injection, its potential DNA binding is either disrupted or if maintained the fraction of DNA-bound RNA is left behind. Hence, injected RNA consists of either previously bound DNA that lacks potential stability or other functionality conferred by DNA binding or is deprived of the RNA fraction usually bound to DNA. This is a clear limitation of prior studies, including our own, and might explain at least in part why a full mimic of phenotypes has seldom been achieved. A concern that adds to this is the often highly complex secondary structure of RNAs, which might be key for stability and function yet could get altered in such experimental approaches. tRFs have complex secondary structures similar to tRNAs and bind with their 5ʹ fragments [121]. Similarly, lncRNAs rely on their secondary structure to bind their targets [122]. The *cis-* and *trans-*acting of lncRNAs in DNA:RNA hybrid triplex formations and their function in gene regulation has been summarized by Li and colleagues. They suggest lncRNAs bind the DNA double helix based on triplex-forming motifs [93].

To circumvent a potential loss of (i) secondary structures and (ii) stability, inhibition of DNA:RNA hybrids by complementary oligonucleotides could provide an elegant approach of reverse paternal phenotypes. A reversal by antisense oligos has been achieved so far by the group of Chen and by Benito *et al.* for miRNAs hence targeting post-transcriptional gene regulation [34, 48]. It remains to be seen whether approaches targeting DNA-bound RNA can be effective too. Targeting bound RNA is inevitably more challenging. Instead of using oligonucleotides, aptamers could be applied to bind on the DNA:RNA triplex. Aptamers are synthetic protein or deoxy-/ribonucleic acid-based small molecules that can bind biological structures by their three-dimensional conformation [123]. They have been established already as small interfering RNA (siRNA) chimeras [124]. Specific aptamers could be synthesized targeting DNA:RNA hybrids or specific DNA sites that usually form triplex structures, prohibiting the attachment of RNA to the DNA. A prevention of behavioural and metabolic phenotypes in the offspring of environmentally exposed males by aptamers would be an unequivocal indication that DNA-bound RNAs indeed are needed for epigenetic germline transmission.

In an alternative approach, recently employed by Jung *et al.* [76], a deletion in the region encoding m6A demethylase, where additionally a specific enhancer RNA (eRNA) would bind, achieved the correction of a BPA-induced phenotype [76].

Overall, the mechanism of how paternal RNA is contributing to embryonic expression in terms of how it is delivered from sperm to oocyte and how it interacts with the embryonic genome is not understood yet. We proposed experiments to dissect the chromatin–RNA interaction as a possible means to understand the mechanism of RNA-mediated epigenetic inheritance.

## Conclusion

The field of epigenetic germline inheritance is vastly expanding, and new studies on paternal influence in the transmission of environmental influences emerge continuously. A so-called “sperm RNA code” was postulated [30] based on many studies demonstrating the influence of distinct RNA classes. It seems clear that all these RNA classes are involved in one way or the other in the transmission of environmental effects to influence the offspring. RNAs are altered through environmental challenges in sperm, either during spermatogenesis or in the epididymis, and delivered to the oocyte during fertilization. Nevertheless, the detailed mechanism remains largely unclear. It is unknown whether paternal RNA indirectly affects embryonic expression by regulation leading to a persistently altered expressive state or whether they persist until later embryonal stages where they directly modulate embryonic gene expression. Furthermore, it is unclear how a given metabolic or psychological stressor leads to changes in RNA payload or RNA modifications mechanistically and whether there are converging RNA signatures of distinctively different exposures. As outlined, several technically highly challenging experimental approaches might help to elucidate how a particular sperm RNA signal is established under specific environmental influences, how it is propagated and how it brings about phenotypic changes in the offspring.

Lastly, to come back to the notion of transgenerational effects, RNA arguably would require a self-perpetuating signal reminiscent potentially of that of the piRNA ping-pong cycle [125]. Alternatively, and potentially more likely, however, the sperm RNA signal is embedded in a complex interplay with other epigenetic modifications, such as seen for Fto enhancer and chromatin states [85]. Such interactions could (i) induce a translation of RNA signals into other more stable modifications in the offspring, (ii) induce another cycle of RNA-mediated inheritance or (iii) even lead to a genetic consolidation by altering the propensity for mutations via RNA-directed methylation [44] or transposable element activity [48, 126].

Lastly, we predict that a more holistic assessment of the interplay of different sperm RNA classes but also other epigenetic marks in combination with gene expression analysis and experimentation targeting reversal as opposed to mimics will substantially contribute to clarifying the mechanistic contribution of sperm RNA to intergenerational and potentially transgenerational effects.

## Data Availability

Not applicable.
